# The functioning of the Cuban home hospitalization programme: a descriptive analysis

**DOI:** 10.1186/1472-6963-7-76

**Published:** 2007-05-31

**Authors:** Pol De Vos, Isabel Barroso, Armando Rodríguez, Mariano Bonet, Patrick Van der Stuyft

**Affiliations:** 1 Department of Public Health, Institute of Tropical Medicine, Antwerp, Belgium; 2 Department of Epidemiology and Health Services, Institute of Hygiene Epidemiology and Microbiology, Havana, Cuba

## Abstract

**Background:**

Over the last decades hospital at home (HaH) programmes have been set up in many, mainly European, countries. The Cuban HaH programme is not hospital driven, but the responsibility of the first line health services, and family doctors play a pivotal role.

**Methods:**

We analyse the structure and functioning of the Cuban programme. In this descriptive study, information was prospectively collected on HaH patients admitted between July 1^st ^2001 and June 30^th ^2002.

**Results:**

Admission rates varied between areas from 0.014 to 0.035 per person per year (ppy). The < 1y and 1–4y age groups had the highest admission rates. In one area the follow-up of pregnancy problems led to high 15–24y and 25–49y female admission rates (0,070 and 0,058 respectively). Respiratory affections were the most frequent reason for admission (32,6%), followed by early hospital discharge (16,0%) and gynaeco-obstetrical problems (10.8%). The median length of stay varied from 5 to 7 days between regions and from 5 days (early discharge) to 7 days (gynaeco-obstetrical problems) in function of the reason for admission. On average an HaH episode entailed 1.4 and 1.6 contacts per patient-day with the family doctor and nurse respectively.

**Conclusion:**

Difference in admission criteria in function of geography, distance to the hospital, transport facilities, and staff factors, as well as differences in hospital policy on early discharge explain the observed variability. The programme plays an important role in the integrated approach to quality care in the Cuban health system, but could benefit from more uniform admission criteria.

## Background

After some initial attempts in the United States and Canada, the concept of hospital at home (HaH) finds its origin in 'Hospitalisation à Domicile' in France in 1951, a service that provides, in the patient's home, treatment by health care professionals for conditions that otherwise would require hospital in-patient care.[1-3] HaH has subsequently been implemented in a number of other, mainly western, countries, but the schemes vary in their philosophy and in the type of care provided. Many countries use the term of 'home care program' instead of hospital-at-home. Although home care programs are likely to cover a broader array of care initiatives, some home care programs provide care to permit early discharge from hospital, and prevent hospital use through home palliative and seniors care. [4] Many are designed for specific conditions or types of care, while some admit a large range of health problems.[5] Essential factors that influence how HaH is being applied are, on the one hand, characteristics of the national health system and its financing mechanisms and, on the other hand, the social environment, the economic situation of the families, and the opportunity for family members to participate in the care for the patient.[6]

A recent Cochrane Review of randomised trials of HaH care compared with hospital in-patient care included 16 randomised trials.[7] Evaluations of obstetric, paediatric and mental health HaH schemes were excluded. All included schemes only admitted patients aged 18 years and over, and 11 only elderly. Three trials concerned patients following elective surgery, two trials analysed patients with a terminal illness, and one included patients with a mix of surgical and medical conditions. The others limited intake to medical conditions. The review concludes that there exists insufficient evidence for claiming a difference in health outcomes or cost to the health service. Allocation to HaH resulted in a reduction in hospital length of stay, but HaH increased overall length of care. Early discharge schemes, for patients recovering from elective surgery and elderly patients with a medical condition, might result in reducing the pressure on acute care hospital beds. Patients allocated to HaH expressed greater satisfaction with the care provided than those in the hospital. Professional caretakers notwithstanding, expressed less satisfaction with the HaH programme.

Unfortunately, there is still a scarcity of studies that examine the concrete place and functioning of HaH within specific socio-economic contexts and health systems and that take into account the characteristics of patients, care organisation, professionals involved, and financing mechanisms.[8] We analyse here the structure and the functioning of the Cuban HaH programme in different geographic areas of the country. In the discussion we focus on understanding the context-specific features and on outlining the scope for improving the effectiveness and efficiency of the scheme.

## Methods

### 1. The Hospital at home programme in the Cuban health system

Cuban municipalities are divided in health areas, in which a policlinic – with on average 30 to 50 family doctors and nurses, 5–10 specialists and the necessary supporting staff – organises the first line health care for about 30.000 inhabitants. A family doctor and a nurse form a basic health team that is responsible for the integrated curative, preventive and promotive health care of a geographically well-defined population of about 700–800 inhabitants. About 15 basic health teams constitute a 'basic working group' (BWG) that covers between 5.000 and 10.000 inhabitants, supported by a paediatrician, a gynaeco-obstetrician, a specialist of internal medicine and a psychologist. Around 3 to 5 BWG are linked to one policlinic and use the diagnostic services (laboratory, medical imaging, etc) and specialist outpatient consultations it provides.[9]

Within the context of a low-income non-market economy, Cuba's national health system is based on the principles of equity, effectiveness, participation, and state responsibility, and is world-wide recognised as effective.[10] In the early 90's, radical changes in the terms of trade with the former Soviet Union and the tightening of the USA blockade drastically reduced the national income. This entailed a 'Cuban style' health sector reform that ratified the principles of state financing of the health care system and of universal coverage and accessibility through free services, while pursuing mayor efficiency.[11] Amongst others, in order to enhance the family doctor programme, reinforced attention was given to a HaH scheme.

The Ministry of Public Health (MINSAP) defines HaH as the intensive follow-up at home (i.e. at least one home visit a day by the doctor and the nurse and more if needed), by the first line health personnel, of patients that require bed rest and/or isolation, without having a compelling technical need for hospital admission. This implies that the health problem is not life threatening and does not require continuous medical supervision, and that all medical examinations, treatment and follow-up must be feasible at home or at the policlinic. Furthermore, the patient and his family must accept HaH, and the socio-economic, hygienic and environmental conditions must be adequate. But the HaH care model is flexible, and in practice also used for ensuring a close follow-up at home of patients that would, without HaH, not necessarily be hospitalised.[12,13]

The Cuban Hospital at home scheme is not a "hospital-driven" model as developed in different European countries, where the hospital staff is in charge of the HaH patients.[14-16] In Cuba, like in the UK, the family doctors are responsible for this programme and admission implies a daily follow-up by the family doctor and nurse, with support of the specialists of the BWG when needed.[17] Furthermore, the HaH scheme covers a wide range of health problems, including paediatric, post-operative, palliative and pregnancy related care.[18]

### 2. Selected study sites

The implementation of the HaH programme differs slightly in function of the setting. Therefore four municipalities were purposely chosen, one in each of the existing environments in Cuba: *Playa*, a metropolitan municipality of the capital Havana; *Cruces*, a small city in the province of Cienfuegos; *Union de Reyes*, a rural community in the Matanzas province; and mountainous *Fomento *in the Santi Espiritus province. In each of these study areas the population covered by one 'basic working group' (BWG) was included.

### 3. Data collection and analysis

Information was collected on all HaH patients admitted between July 1st 2001 and June 30^th ^2002, for which all appropriate ethical approvals were obtained from the ethical review commission of the Cuban and Belgian research institutes. A specific form was designed and variables recorded included patient characteristics, date of HaH admission and discharge, reason for admission, number and duration of patient-staff contacts, and outcome. Local health staff that previously had been trained in the use of the forms registered all data concomitant to their daily activities in the HaH programme. In each municipality a project co-ordinator ensured supervision and monitored the quality of the data collection and data entry. Statistical analysis was carried out with SAS 8.0.[19]

## Results

Over the study period, a total of 837 HaH cases were registered in the 4 study areas (Table 1). There was important variation in the HaH rates between the different Basic Working Groups (BWG) involved in the study, from 0.014 HaH cases per person per year in Playa to 0.035 in Fomento.

**Table 1:** Hospital at home admissions and family doctor contacts by study area (July 2001 – June 2002)

	**U. de Reyes**	**Cruces**	**Fomento**	**Playa**
*Hospital at home admissions*				
**Number**	225	287	190	135
**Population**	8247	10759	5513	9481
**Rate (ppy)**	0,027	0,027	0,035	0,014
*Family doctor contacts*				
Total contact rate (ppy) (*)	8,4	4,1	3,2	3,1
**HaH specific contact rate (ppy)**	0,176	0,170	0,483	0,095
**HaH contacts as % of total F.Dr. contacts**	2,1%	4,1%	15%	3%
Average number per HaH episode	6,5	6,3	13,8	6,8

(ppy) = per person and per year

(*) = total of preventive and curative family doctor contacts

Table 2 gives the sex and age specific rates for each of the study areas. Rates were markedly higher for infants. In the age groups 15–24y and 25–49y, women had systematically higher admission rates than men, particularly in Fomento.

**Table 2:** Hospital at home admissions: sex and age specific numbers and rates (per person year) by study area (July 2001 – June 2002)

	**Union de Reyes**				**Cruces**				**Fomento**				**Playa**			
	*Males*		*Females*		*Males*		*Females*		*Males*		*Females*		*Males*		*Females*	

**Age group**	***N***	***Rate***	***N***	***Rate***	***N***	***Rate***	***N***	***Rate***	***N***	***Rate***	***N***	***Rate***	***N***	***Rate***	***N***	***Rate***
***< 1 y***	23	0,479	26	0,426	26	0,295	27	0,325	10	0,357	4	0,174	5	0,152	5	0,100
***1 – 4 y***	19	0,092	17	0,098	28	0,103	16	0,070	4	0,032	4	0,028	11	0,061	13	0,062
***5 – 14 y***	14	0,024	9	0,018	6	0,008	10	0,015	10	0,027	3	0,008	10	0,019	4	0,008
***15 – 24 y***	3	0,006	8	0,017	3	0,004	13	0,021	7	0,019	23	0,070	2	0,004	5	0,012
***25 – 49 y***	8	0,005	26	0,018	21	0,011	54	0,028	19	0,016	63	0,058	6	0,003	26	0,015
***50 – 59 y***	5	0,010	5	0,010	7	0,011	8	0,011	6	0,021	6	0,021	5	0,007	6	0,010
***> 60 y***	32	0,036	30	0,037	34	0,037	34	0,029	15	0,030	16	0,041	12	0,010	25	0,027
**Total**	104	0,024	121	0,030	125	0,024	162	0,030	71	0,025	119	0,045	51	0,010	84	0,019

Respiratory problems were the most frequent reason for HaH admission (Table 3) and (broncho)pneumonia represented 13% (108/837) of all diagnoses. It was the most frequent diagnosis for HaH in men (24%), and in the +60y age group (31%). A broader array of respiratory infections (including bronchitis, bronchopneumonia and pneumonia) was the most common reason for HaH in the age groups < 1y and 1–4y, accounting for 50% and 45,5% respectively of the admissions in this age groups. In Fomento the rate for gynaeco-obstetrical problems was to 3 times higher than in other areas due to much higher admissions for the monitoring of pregnancy and delivery complications, which made up 20% of all admissions. A total of 134 patients, 60% women, were admitted in HaH after an early hospital discharge, frequently (but not exclusively) related to a surgical intervention. Specific rates were higher in Cruces and Fomento. Finally, also 32 HaH patients were included in the programme to ensure palliative care for a terminal illness. In this group, 60% were men and 89% was more than 60 years old.

**Table 3:** Most important reasons for admission in the HaH programme by study area (July 2001 – June 2002)

	***Total***	**Union de Reyes**	**Cruces**	**Fomento**	**Playa**
**Reason for admission**	***Number***	**Number**	**Number**	**Number**	**Number**

*Respiratory problem*					
(Broncho)pneumonia	*108*	47	31	6	24
High respiratory infection	*103*	10	48	22	23
Bronchitis and COPD	*36*	16	4	14	2
Asthma	*15*	2	5	5	3
Other	*11*	0	5	3	3
**Total**	***273 (33%)***	**75**	**93**	**50**	**55**
					
*Gynaeco-Obstetrical problem*					
Pelvic Inflammatory Disease	*11*	1	9	1	0
Complications of pregnancy	*63*	6	17	30	10
Complications of postpartum	*16*	5	0	7	4
**Total**	***90 (11%)***	**12**	**26**	**38**	**14**
					
*Gastro-intestinal problem*					
Intestinal infections	*53*	24	17	2	10
Hepatitis	*11*	0	8	1	2
Gastric and duodenal ulcer	*4*	2	0	2	0
**Total**	***68 (8%)***	**26**	**25**	**5**	**12**
					
*Cardiovascular problem*					
Unstable hypertension	*23*	1	8	11	3
Ischemic heart disease	*5*	0	5	0	0
Chronic heart insufficiency	*9*	5	1	0	3
Peripheral circulatory problems	*21*	4	4	11	2
Others and unspecified	*7*	0	6	0	1
**Total**	***65 (8%)***	**10**	**24**	**22**	**9**
					
*Early hospital discharge*	***134 (16%)***	**17**	**77**	**26**	**14**

**Figure 1:**
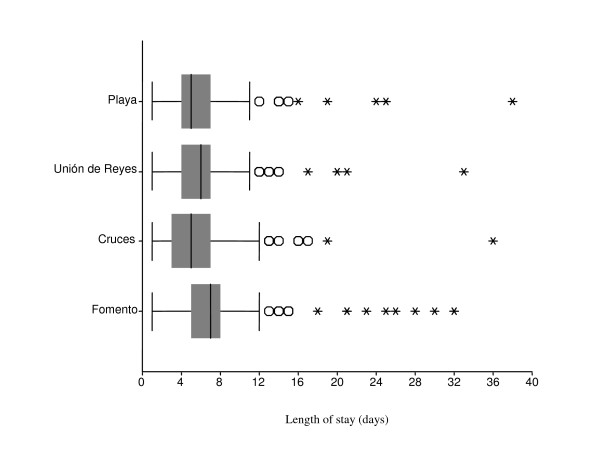
**Length of stay in the Hospital at home programme by study area (July 2001 – June 2002)**. The boxplot diagram shows the median, interquartile range, 1,5 × interquartile range, and outliers. Three observations are omitted (Fomento), with a duration of 67 days, 82 days, and 97 days.

**Figure 2:**
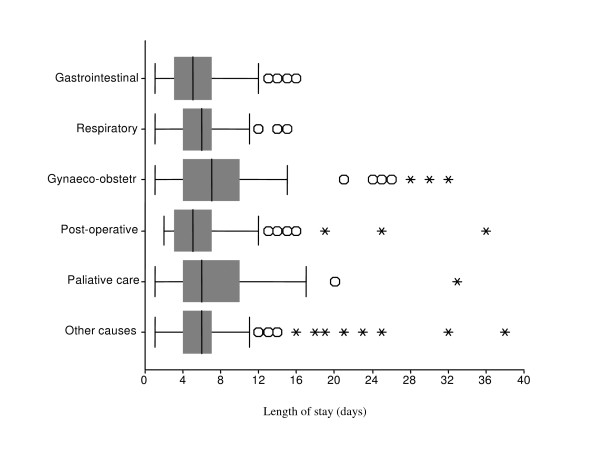
**Length of stay in the Hospital at home programme for frequent reasons for admission (July 2001 – June 2002)**. The boxplot diagram shows the median, interquartile range, 1,5 × interquartile range, and outliers. Two gynaeco-obstetrical observations (67 and 82 days) and one palliative care observation (97 days) are omitted (Fomento).

The median length of stay in HaH in Fomento (7 days (CI 95% = 6.77; 7.23)) was significantly higher than in Playa (5 days (4.37 – 5.63)), Cruces (5 days (4.60 – 5.40)) and Union de Reyes (6 days (5.50; 6.50)) (Figure 1). Some outliers are note worthy. In Fomento, two pregnant women were in the programme for 67 and 82 days, and one palliative care patient for 97 days. The box-plot in Figure 2 represents the distribution of the length of stay for the most frequent reasons for admission. The median length of stay for early discharge HaH episodes (5days (4.36; 5.64)) was significantly shorter than for respiratory diseases (6days (5.71 – 6.29)) and gynaecological admissions (7 days (5.99 – 8.01)); there was no significant difference with palliative care (6 days (4.99 – 7.01)), gastrointestinal affections (5 days (3.95 – 6.05)) and 'other causes' (6 days (5.57 – 6.43)). The median length of stay for palliative care showed the widest variation (from 1 to 97 days).

The family doctor and nurse had on average of 8.2 and 9.3 patient contacts per HaH episode respectively or on average 1,4 and 1.6 contacts per patient day, but there was important variation between areas. In Fomento, where the average length of stay was longer, there were on average 13.8 doctor visits per episode, while in the other areas the average lay between 6,3 and 6,8 visits per episode. In Fomento, family doctors also had the highest number of HaH patient contacts per person per year and the lowest overall number of patient contacts per person per year, resulting in a very high proportion of 15% for HaH related contacts; all other areas had proportions between 2,1% and 4,1% (Table 1). No differences in average number of patient contacts were found in function of the reason for admission. On average, the family doctor spent a total of 175 minutes per patient admitted in the programme, or 22 minutes per visit. The average total time spent by the nurse was 185 minutes per HaH admission (20 minutes per visit).

Supporting specialists from the Basic Working Group to which the family doctor belongs, had an average of 0.7 visits per patient (varying between 1.3 visits in Union de Reyes and 0.2 visits in Cruces) with an average duration of 35 minutes per visit. Other specialist staff – i.e. not related to the BWG – was involved in the follow up of HaH patients with on average 0.1 visits per patient (between 0.3 in Union de Reyes and 0.01 in Cruces) of on average 40 minutes duration. With regard to technical examinations, radiography was required in 118 patients, mainly the ones with respiratory diseases (72%). Electrocardiography was realised in 27 patients, while laboratory tests were needed for 71 patients. For some technical interventions the patient had to go to the nearby polyclinic. Depending on the patient's condition and on the availability of the ambulance at the polyclinic, this transfer was organised by the health services.

Finally, 92.9% of the 837 patients were discharged from the HaH programme in good health, while 23 (2.7%) died and 37 (4.4%) were referred to the hospital. Of the latter, 16 referrals (43%) were for causes related to pregnancy and delivery, while the others were for various types of deterioration of the health status of the patient. All deceased were terminal patients under palliative care.

## Discussion

The diagnostic pattern in the HaH programme in Cuba is comparable to the one observed in Spain, where respiratory tract affections and gastrointestinal illnesses are also the main causes for admission.[20,21] However, it is completely different from what is observed in the United Kingdom, where orthopaedic problems and palliative care are most frequent, while in France and the United States, cancer and circulatory affections rank first.[22] These differences are related to the design of the respective programmes, their priorities and admission criteria, and of course also to a conceptual difference: in all the mentioned countries the HaH programme is under responsibility of the hospital.

Whereas a hospital based program is mainly reactive to illness and has a periodic care concept, the Cuban HaH programme is not solely designed as an alternative for or an extension of hospital admission. It also covers different aspects of the broader concept of 'home care programs' as they exist in other countries (e.g. Canada), ensuring and documenting a close follow-up by the first line health team when daily home visits are needed in ambulatory care.[4,23] This kind of follow-up by first line health workers is not contemplated in hospital based HaH programmes.

Precise criteria for admission to the programme inside countries can furthermore differ in function of the local setting. In Cuba, the geography (distance and access to the hospital, urban or rural environment), transport facilities (availability of public transport or of an ambulance), staff factors (organisation of the BWG and included specialists, motivation), etc... partly explain the variability in the overall and specific admission rates between the different study areas. Playa, with the lowest overall HaH admission figures, is surrounded by an array of metropolitan hospitals. Fomento, with the highest overall rates, is mountainous and has, due to its relative geographical isolation, a more difficult access to the hospital. For the same reason, a higher proportion of pregnant women in this area is admitted to HaH for the follow-up of at risk pregnancies; the director of the municipal health services being a very motivated gynaecologist further contributes to the emphasis put on this part of the HaH programme.

Similarly, Unión de Reyes has a quite high under-five HaH admission rate. The fact that the reference hospital is located at a distance of 60+ km, and the municipal health director being a very dedicated paediatrician play here an important role. Hospital policy on early discharge, on the other hand, is the mayor determinant for the wide variation in early discharge HaH rates, which are highest in Cruces and in Fomento. Still, the observed variations in the admission pattern to the HaH programme between the different areas involved in the study can only partially be explained by context variables, of which some are, furthermore, of rather personal nature. These important variations could suggest that there is room for some more uniformity in the admission criteria between the different settings, without loosing the scope for adaptations to local circumstances. Also, clearer rules for early hospital discharge and a better co-ordination between the first and second line services could ensure a more adequate development of the corresponding component of the HaH programme.

Fomento has the longest average length of stay for HaH episodes. The high number of pregnancy related admissions contributes to this: These risk based admissions, by their preventive nature, entail longer lengths of stay. A further explanation is the dispersion of the population in this mountainous area: the more difficult access to health services motivates the family doctor not to discharge patients from the HaH programme before they are fully cured. In all areas the variation in the length of stay for palliative care patients is pronounced, with some patients leaving the hospital only a few days before passing away, while others are being cared for at home for months. More than for any other reason for admission, this variation is what can be expected, as the scope for palliative home care largely depends on the social environment and the carrying capacity of the family. In general single persons and those who do not have families are at greater risk of hospitalization, since families appear (as they do in all countries) to be the consistent daily caregivers for their ill family members.

To estimate the share of workload attributable to the HaH programme, we included in the numerator of the total family doctor contact rate all preventive as well as curative contacts in the practice and at home. Preventive and curative home visits – independent from the HaH programme – are, indeed, central to the Cuban family medicine concept and part of the daily activities of family doctors and nurses consists of visiting families they are responsible for. Eventually, the HaH programme has only a limited impact on the overall workload, except in Fomento where, for the reasons discussed above, the number of family doctor contacts per HaH episode is considerably higher.

Doctors and nurses have comparable average number and average total duration of contacts with HaH patients since – most of the time – they organise joint visits. This ensures of course good co-ordination, but is also symptomatic for the lack of a clear role description for the nurse in the HaH programme. A better definition of his/her specific tasks and individual responsibilities would enhance efficiency by diminishing the workload, and boost professional satisfaction.

Free health care is an important pillar of the Cuban social system. However, HaH programmes can induce extra costs for a family that cares for a patient.[24] Cuban health authorities are aware of this pitfall and try to avoid it: Except for drugs (free in the hospital, heavily subsidised at the first line) the direct costs do not differ between hospital and home hospitalisation.

Finally, in contrast to what has been concluded from a review of HaH schemes under hospital responsibility,[7] the Cuban first line driven scheme could assure increased satisfaction of all actors involved, including the medical and paramedical staff.

More individualised care organised at the patient's home by the family doctor and nurse permits to develop a more horizontal health staff – patient relation, in which most of the diagnostic and therapeutic decisions are negotiated between the doctor, the patient and his family.[25,26] Furthermore, the family can play an essential role to ensure adequate treatment, follow-up, care and/or rehabilitation.[27,28] This eventually leads to more effective and efficient integrated care.

## Conclusion

Difference in admission criteria in function of geography, distance to the hospital, transport facilities, and staff factors, as well as differences in hospital policy on early discharge explain the observed variability. The programme plays an important role in the integrated approach to quality care in the Cuban health system, but could benefit from more uniform admission criteria.

This Cuban experience, and in particular its design and the central role of the first line health services in it, is of relevance for other developing or industrialised countries who plan to set up or reorient hospital at home programmes from reactive hospital-based systems to a comprehensive programme embedded in the first line care level.

However, given the current trends in health sector reform worldwide and the alternative direction taken by Cuban policy makers, organisational particularities might be specific to the local context.

## Competing interests

The author(s) declare that they have no competing interests.

## Authors' contributions

PDVparticipated in the conception, the design and the implementation of the research, in the analysis and interpretation of the information, and in the drafting and revision of the paper. IBparticipated in the design of the research, in the analysis and interpretation of the information, and in the drafting and revision of the paper. AR participated in the design and the implementation of the research, and in the drafting and revision of the paper. MB participated in the conception, design and implementation of the research, and in the drafting and in the revision of the paper. PVdS participated in the conception and the design of the research, in the interpretation of the information, and in the drafting and revision of the paper.

## Pre-publication history

The pre-publication history for this paper can be accessed here:


